# The *Mycoplasma hyopneumoniae* protein Mhp274 elicits mucosal and systemic immune responses in mice

**DOI:** 10.3389/fcimb.2025.1516944

**Published:** 2025-02-07

**Authors:** Mengqi Xie, Zhongshun Huang, Yun Zhang, Yujie Gan, Huiying Li, Dan Li, Honglei Ding

**Affiliations:** Laboratory of Veterinary Mycoplasmology, College of Veterinary Medicine, Southwest University, Chongqing, China

**Keywords:** mycoplasma hyopneumoniae, IgG, IFN-γ, IL-4, IL-17, lymphocyte proliferation response

## Abstract

**Background:**

*Mycoplasma hyopneumoniae* is the etiological agent of mycoplasmal pneumonia of swine (MPS). Commercial vaccines provide partial protection and do not prevent the colonization and transmission of *M. hyopneumoniae*. The bottleneck in the development of more effective vaccines for MPS is the stimulation of effective immune responses in the host. The purpose of the present study was to evaluate the immune responses of immunodominant proteins Mhp170, Mhp274 and Mhp336 in BALB/c mice.

**Methods:**

The recombinant Mhp170 (rMhp170), Mhp274 (rMhp274), and Mhp336 (rMhp336) proteins were purified from recombinant bacteria. Fifty-two six-week-old SPF female BALB/c mice were divided into five groups: a commercial inactivated vaccine-immunized group, three recombinant protein-inoculated groups, and a PBS-treated group. The physical parameters and body weights of the mice were observed during the experiment. The lung/body coefficient and macroscopic and microscopic lung lesions were evaluated. IgG and its isotypes IgG1 and IgG2a in serum and BALF and sIgA in BALF were assessed. The levels of IFN-γ, IL-4, and IL-17, in the supernatants of splenocytes and in serum were measured, and the mRNA levels of three cytokines in splenocytes were analyzed. Finally, lymphocyte proliferation after stimulation with corresponding proteins or crude extract of *M. hyopneumoniae* J strain was assessed.

**Results:**

We successfully constructed recombinant bacteria expressing rMhp170, rMhp274, and rMhp336. None of the mice from all groups presented adverse reactions and macroscopic and microscopic lung lesions. rMhp170 and rMhp274 were capable of inducing the production of IgG, IgG1 and IgG2 in serum and BALF, the secretion of IFN-γ, IL-4 and IL-17 in serum, the expression of IFN-γ, IL-4 and IL-17 mRNAs in splenocytes, and high levels of lymphocyte proliferation. Moreover, rMhp274 significantly increased sIgA in BALF. Nevertheless, rMhp336 induced only IgG, IgG1 and IgG2 production in sera; the secretion of IFN-γ and IL-4 in sera and BALF; the expression of IFN-γ and IL-4 mRNAs in the splenocyte population; and lymphocyte proliferation.

**Conclusion:**

Mhp170 and Mhp274 induced Th1/Th2/Th17 immune responses, and Mhp336 stimulated mixed Th1/Th2-type immune responses, in mice. Our data suggest that Mhp274 is a potential viable candidate for the development of a subunit vaccine for MPS.

## 1 Introduction

*Mycoplasma hyopneumoniae* is the etiological agent of mycoplasmal pneumonia of swine (MPS), which is also referred to as enzootic pneumonia (EP) (Almeida et al., 2023; Ferraz et al., 2020; Siugzdaite et al., 2003). This disease, characterized by chronic nonproductive coughing and decreased daily weight gain, causes massive economic losses to the swine industry worldwide (Almeida et al., 2023; Ferraz et al., 2020) and facilitates secondary infection by other pathogens, such as *Glaesserella parasuis*, *Actinobacillus pleuropneumoniae*, *Pasteurella multocida*, *Streptococcus suis*, porcine circovirus type 2, and porcine reproductive and respiratory syndrome virus (Renzhammer et al., 2023). The pathogen, which colonizes the lower respiratory tract, causes the formation of lung lesions characterized by variably sized areas of dark consolidation in the apical, cardiac, and intermediate lobes (Toledo et al., 2023).

MPS prophylaxis involves the administration of antimicrobials, the implementation of management protocols, and vaccination (Maes et al., 2018). Vaccination with attenuated live and inactivated adjuvanted vaccines, which are frequently used worldwide to control *M. hyopneumoniae* infection (Maes et al., 2021; Hao et al., 2022a), is considered the most cost-effective method for decreasing clinical signs and lung lesions, decreasing mortality, reducing the localization of the organism, and improving production performance (Maes et al., 2021). Mucosal and cellular immune responses play important roles in protection against *M. hyopneumoniae* (Ferreira et al., 2023; Petri et al., 2024; Rodríguez et al., 2023). Several studies have shown that secretory immunoglobulin A (sIgA) prevents pathogens from adhering to the respiratory tract, whereas IFN-γ production enhances phagocytosis by alveolar macrophages (Almeida et al., 2023; Ferreira et al., 2023; Rodríguez et al., 2023). However, inactivated vaccines have certain restrictions related to antigen presentation pathways, resulting in a limited mucosal immune response, which requires adjuvants or immunostimulants to enhance the response (Martelli et al., 2014). Some studies have demonstrated that inactivated vaccines only provided uncertain beneficial effects (Okada et al., 1999; Villarreal et al., 2011a), as lesions were found to be present in the lungs of pigs infected with *M. hyopneumoniae* after immunization with vaccines (Michiels et al., 2017), the daily weight gain was not improved significantly (Meyns et al., 2006; Villarreal et al., 2011a), and the colonization in the lung and transmission of *M. hyopneumoniae* in vaccinated group could not be prevented (Meyns et al., 2006; Villarreal et al., 2011a, 2011b). The attenuated *M. hyopneumoniae* vaccine (strain 168) is licensed and widely used in China. However, immunization with the strain 168 vaccine induces the overexpression of IL-10 and the downregulation of IFN-γ expression, suggesting that vaccination with strain 168 alone cannot achieve productive immune effects in practical applications (Shen et al., 2017). Moreover, owing to the complex medium requirements of mycoplasmas, the production costs of these commercial vaccines are expensive (Cook et al., 2016). Therefore, a new type of vaccine that can induce durable mucosal, humoral, and cellular immune responses against *M. hyopneumoniae* in pigs at a lower cost and can achieve complete protection is urgently needed. As a result, vaccine development strategies have shifted toward subunit-based vaccines, which have become a viable alternative.

Several immunodominant and immunoprotective proteins of *M. hyopneumoniae* have been identified, including P97 (Liu et al., 2023; Wang et al., 2019; Zhou et al., 2022), P42 (Galli et al., 2012; Jorge et al., 2014; Zhou et al., 2022), P46 (Galli et al., 2012; Liu et al., 2023; Wang et al., 2019), P95 (Galli et al., 2012), Mhp390 (Liu et al., 2023), nicotinamide adenine dinucleotide-dependent (NADH) flavin oxidoreductase (NFPR) (Xie et al., 2021; Xu et al., 2022) and NADH oxidase (NOX) (Hao et al., 2022b; Xu et al., 2022). These proteins have been widely used to develop subunit vaccines, and some of these proteins provide partial protection against *M. hyopneumoniae* infection by inducing immune responses to varying degrees. For example, a chimeric vaccine assembled with the recombinant protein LTB-P97R1-Mhp390-P46 elicited significant cellular immune responses and increased the production of IgG and IgM antibodies against *M. hyopneumoniae* in mice (Liu et al., 2023). Following challenge with virulent *M. hyopneumoniae*, pigs orally immunized with attenuated *Salmonella* Typhimurium aroA SL3261 expressing the recombinant antigen NrdF presented greater average daily weight gains and fewer lung lesions than the control groups did (Fagan et al., 2001). P42 and P95, which have been used as subunit vaccines by mixing with aluminum hydroxide adjuvant, have been shown to induce high expression of IFN-γ and the production of IgG antibodies in mice (Galli et al., 2012). Recombinant *M. hyopneumoniae* heat shock protein P42 emulsified in an oil-based adjuvant triggered both cellular and humoral immune responses in piglets (Jorge et al., 2014). However, antigens that can stimulate the production of mucosal, and systemic immune responses simultaneously may exhibit better immune protection against *M. hyopneumoniae* infection.

Previous research revealed several humoral immunodominant proteins that can strongly react with *M. hyopneumoniae*-positive porcine sera, including three hypothetical proteins, Mhp170, Mhp274 and Mhp336 (Ning et al., 2020, 2023). In other words, these proteins have the potential for use as candidate antigens for the development of new vaccines. The purpose of the present study was to evaluate the capacity of Mhp170, Mhp274 and Mhp336 to elicit mucosal, humoral, and cell-mediated immune responses in mice. Our aim was to identify protein antigens that may be applied in the development of vaccines against *M. hyopneumoniae* infection.

## 2 Materials and methods

### 2.1 Bacterial strains and plasmids

*Escherichia coli* BL21(DE3)-pGEX-6P-1-mhp170 (Ning et al., 2023), *E. coli* BL21(DE3)-pGEX-6P-1- mhp274 (Ning et al., 2023) and *E. coli* BL21(DE3)-pET-32a(+)-mhp336 (Gan et al., 2024) strains were constructed and preserved previously in our laboratory. Competent *E. coli* DH5α and *E. coli* BL21(DE3) cells were prepared and preserved at -80°C. The expression plasmid vectors pET-30a(+) and pET-32a(+) were preserved by our laboratory. The *M. hyopneumoniae* J strain was stored in our laboratory and cultivated in KM2 medium (Tuopu, Zhaoyuan, Shandong, China) supplemented with 20% porcine serum (Jianglai, Shanghai, China) at 37°C. Commercial inactivated vaccine (strain J) was purchased from Laboratorios HIPRA (Avda. La Selva, Spain).

### 2.2 Protein expression and purification

On the basis of the gene sequences of *mhp170* and *mhp274* from the *M. hyopneumoniae* 232 strain, two pairs of primers were designed and synthesized (**Supplementary Table S1**). The target genes were amplified with PrimeSTAR^®^ Max DNA polymerase (Takara, Beijing, China) via PCR by using the genomic DNA of *E. coli* BL21(DE3)-pGEX-6P-1-mhp170 and *E. coli* BL21(DE3)-pGEX-6P-1- mhp274 as the templates. The amplified fragments were digested by *Bam*HI and *Xho*I and then inserted into the plasmid vector pET-30a(+) or pET-32a(+). The recombinant plasmids were sequentially transformed into competent *E. coli* DH5α and *E. coli* BL21(DE3) cells via the heat shock method, and *E. coli* BL21(DE3)-pET-30a(+)-mhp170 and *E. coli* BL21(DE3)-pET-32a(+)-mhp274 were constructed to express recombinant proteins Mhp170 and Mhp274. *E. coli* BL21(DE3)-pET-32a(+)-mhp336 preserved in our laboratory (Gan et al., 2024) was used to express recombinant protein Mhp336 directly.

The expression of the three 6 × His-tagged recombinant proteins was induced in the recombinant bacteria harboring pET vectors with 1 mmol/L isopropyl β-D-1-thiogalactopyranoside (IPTG), and the cells were further cultured at 16°C for 20 h on a shaker at 200 r·min^-1^. Then, the expression and expression form of the target proteins in the recombinant bacteria were examined via 12% sodium dodecyl sulfate−polyacrylamide gel electrophoresis (SDS−PAGE) and Western blotting with a mouse anti-His tag antibody (Bioss, Beijing, China). The soluble recombinant proteins were purified with Ni-NTA HisTrap™HP (Cytiva, Shanghai, China), and the concentration of the purified recombinant proteins was measured via a BCA protein assay kit (Epizyme Biotech, Shanghai, China) according to the manufacturer’s instruction.

### 2.3 Ethics statement and immunization of mice

Fifty-two five-week-old specific pathogen-free (SPF) female BALB/c mice were purchased from Chengdu Yaokang Biotechnology Co., Ltd., and maintained under SPF conditions. The mice were acclimated to the new environment under SPF conditions for one week to prevent the impact of transport stress on the experiment. The animal experimental procedures were approved by the Institutional Animal Care and Use Committee of Southwest University (IACUC No. IACUC-20231228-04), and the experiments were performed in accordance with the recommendations in the Guide for the Care and Use of Laboratory Animals of the Ministry of Health, China.

**Figure 1:**
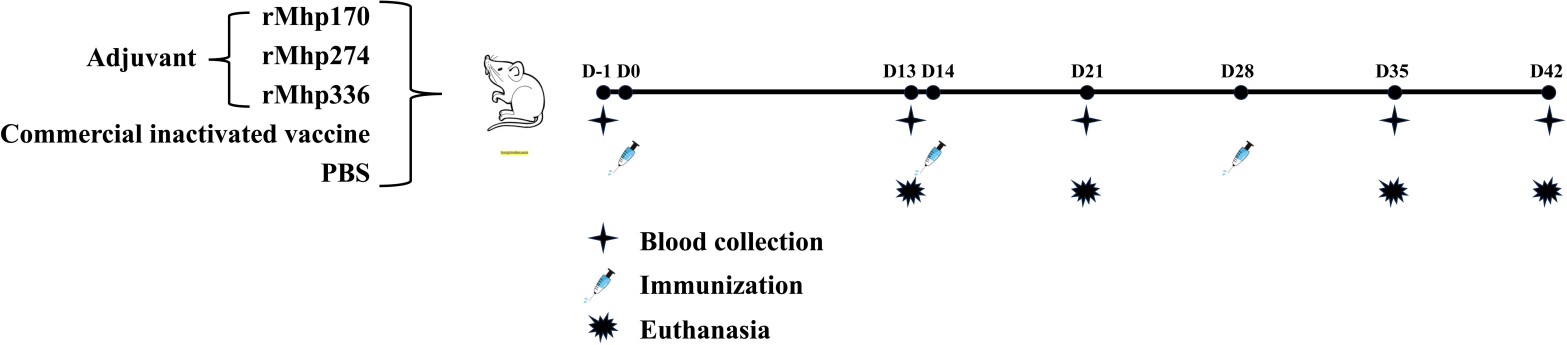
Schematic diagram of the immunization experiment in mice. Fifty-two SPF BALB/c mice were randomly divided into five groups. The mice were immunized subcutaneously with three purified *M. hyopneumoniae* recombinant proteins (12 per group) emulsified with adjuvant three times. The NC and commercial vaccine groups (8 per group) were used as controls.

The experimental scheme is shown in **Figure 1**. At 43 days of age, the mice were randomly divided into five groups as follows: each mouse from group 1 received 0.1 mL of PBS intramuscularly as a negative control (NC, 8 mice); each mouse from group 2 was immunized intramuscularly with 3.4 μL (each bottle of vaccine was diluted to 2 mL) of commercial inactivated vaccine as the positive control (8 mice); and each mouse from groups 3, 4 and 5 was administered 0.1 mL (0.1 mg/mL) of one of the purified proteins emulsified with isometric Freund’s complete adjuvant (BioFROXX, Beijing, China) via multipoint subcutaneous injection into the groins and backs of each group (12 mice). After the first immunization, each mouse from the experimental groups received a booster with the same dose of purified protein emulsified with an equal volume of Freund’s incomplete adjuvant at 14 and 28 days post the first inoculation (DPI). The animal experiment lasted for 6 weeks until all the mice were euthanized.

The status of each mouse, including its appearance, behavior, food and water intake, and survival and death, was examined daily for 5 min after immunization. Moreover, after the first injection, the body weights of the mice in all the groups were measured every 3 days. The body weights and the average weight gain rates (AWGRs) were calculated and compared among the groups.

### 2.4 Necropsy and sample collection

Blood samples were collected from the retro-orbital sinus of each mouse on the day before immunization and at 13, 21, 35, and 42 DPI. Moreover, bronchoalveolar lavage fluid (BALF) samples were randomly collected from two mice in the NC group, two in the commercial vaccine group, and 3 in each experimental group. The mice were then humanely euthanized and necropsied. The whole-lung weight was obtained, and the lung wet weight (g)/body weight (g) ratio (lung/body coefficient) was calculated. The serum samples and BALF samples were stored at -80°C until use.

### 2.5 Macroscopic and microscopic evaluation of lung lesions

Gross lesions of each lung were examined and scored on a scale of 0–3. A score of 0 indicated no damage to the lung, a score of 1 indicated mild damage and minimal consolidation, a score of 2 indicated moderate damage and significant consolidation, and a score of 3 indicated severe damage and overall consolidation of the lung. For histological analysis, lung samples were fixed in neutral buffered formalin, embedded in paraffin, and cut into 3 mm-thick sections. Then, the sections were stained with H&E in a routine histopathological examination. The samples were scored via light microscopy, and the scores ranged from 0 to 3, according to the lesion size and the severity of lymphoid tissue hyperplasia, as described previously (Zhou et al., 2022).

### 2.6 Assessment of immune responses by indirect enzyme-linked immunosorbent assay (ELISA) in serum and BALF

An indirect ELISA was used to analyze the IgG, IgG1 and IgG2a antibodies in the serum, the IgG, IgG1, IgG2a and IgA antibodies in the BALF against the recombinant proteins, and the crude extract of the J strain, as described previously with minor modifications (Liu et al., 2023; Wang et al., 2019; Zhou et al., 2022). Briefly, 100 μL of rMhp170, rMhp274, rMhp336, or a crude extract of *M. hyopneumoniae* J at a concentration of 2.0 μg/mL dissolved in 0.05 mol/L carbonate-bicarbonate buffer (pH 9.6) was used to coat each well of 96-well microtiter plates at 37°C for 1 h and then at 4°C overnight. After washing with 200 μL of PBST (PBS containing 0.05% Tween-20) five times, the plates were blocked with 5% skim milk diluted with PBS at 37°C for 2 h. Then, the plates were washed with PBST five times, and 100 μL of serum (diluted 1:200 in blocking buffer) or BALF (diluted 1:50 in blocking buffer) was added to each well. The plates were then incubated at 37°C for 1 h. Then, the plates were washed five times with PBST, and 100 μL of 1:5000 diluted goat anti-mouse IgG (Bioss, Beijing, China), 1:2000 diluted goat anti-mouse IgG1 (Bethyl Laboratories, Montgomery, TX, USA), IgG2 (Bethyl Laboratories, Montgomery, TX, USA), or IgA (Bethyl Laboratories, Montgomery, TX, USA), which were all conjugated with HRP, was added to each well, and the plates were further incubated at 37°C for 1 h. After washing, 50 μL of substrate A and substrate B (Tian et al., 2021) was added to each well for color development for 15 min at 37°C, and then the reaction was stopped by the addition of 50 μL of 2 mol/L H_2_SO_4_. The optical density (OD) of the plates was read at 450 nm with an ELISA plate reader (Thermo Fisher Scientific, Ratastie 2, FI-01620 Vantaa, Finland). All samples were assayed in triplicate. The cutoff value for positivity was 2.1 times the mean OD_450_ of negative control serum.

### 2.7 Cytokine measurement in mouse culture supernatants of splenocytes and sera

The mouse splenocytes were cultured in 24-well plates at a density of 6 × 10^6^ cells/well with RPMI 1640 medium supplemented with 10% fetal bovine serum (Corning, Woodland, CA, USA) in a humidified incubator with 5% CO_2_ at 37°C. The cells were stimulated with 25 μL of the corresponding purified protein (rMhp170, rMhp274, or rMhp336), the crude extract of the J strain or culture medium (negative control) at a concentration of 10 μg/mL. The supernatant was harvested at 72 h post stimulation and clarified by centrifugation to remove the cell debris. The levels of IFN-γ, IL-4 and IL-17 were measured with corresponding ELISA kits (Huijia Biotechnology, Xiamen, Fujian, China). The cells were collected from each well to extract the total RNA with a Tissue & Cell RNA Extraction Kit (Accurate, Changsha, Hunan, China), and the mRNA expression of specific IFN-γ, IL-4 and IL-17 genes was assessed by quantitative real-time PCR (qRT−PCR) (Wang et al., 2019; Liu et al., 2020) after reverse transcription with a cDNA synthesis kit (Takara, Beijing, China). The primers used for cytokine and control gene amplification are listed in **Supplementary Table S2**. Relative quantification and analysis of IFN-γ, IL-4 and IL-17 cDNA compared with β-actin cDNA with the SYBR Green Premix Pro Taq HS qPCR Kit (Accurate, Changsha, Hunan, China) was conducted on a CFX ConnectTM Real-Time system (Bio-Rad). Each sample in the assay was examined in triplicate.

### 2.8 Spleen lymphocyte proliferation response assay

An assay was used to determine the effects of the recombinant proteins on the cellular immune response. Firstly, spleen lymphocytes were isolated from mouse spleens with mouse lymphocyte separation medium (Solarbio, Beijing, China) under sterile conditions. Then, the splenic lymphocyte suspension was plated in triplicate in 96-well plates at a concentration of 5 × 10^5^ cells/well with RPMI 1640 medium supplemented with 10% fetal bovine serum and maintained in a humidified incubator with 5% CO_2_ at 37°C. The cells were stimulated with 5 μL of the corresponding purified protein (rMhp170, rMhp274, or rMhp336) at a concentration of 10 μg/mL, the crude extract of the J strain (10 μg/mL) or culture medium at 37°C for 72 h. At the end of incubation, 10 μL of CCK-8 medium was added to each well without discarding the culture medium, and the cells were cultured for another 4 h. The OD_450_ was measured with an xMark™ microplate spectrophotometer (Bio-Rad). The stimulation index (SI) values were calculated as the ratio of the OD_450_ of the antigen-stimulated wells to that of the unstimulated wells.

### 2.9 Statistical analysis

All the experiments were performed at least three times. The data were plotted via GraphPad Prism (version 8.0). The data are expressed as the mean ± standard deviation (SD). Multiple group comparisons were performed via one-way ANOVA. The level of significance is indicated as * (*p* ≤ 0.05), ** (p ≤ 0.01), or *** (*p* ≤ 0.001), and *p* > 0.05 was considered statistically nonsignificant.

## 3 Results

### 3.1 Recombinant protein expression and purification

**Figure 2:**
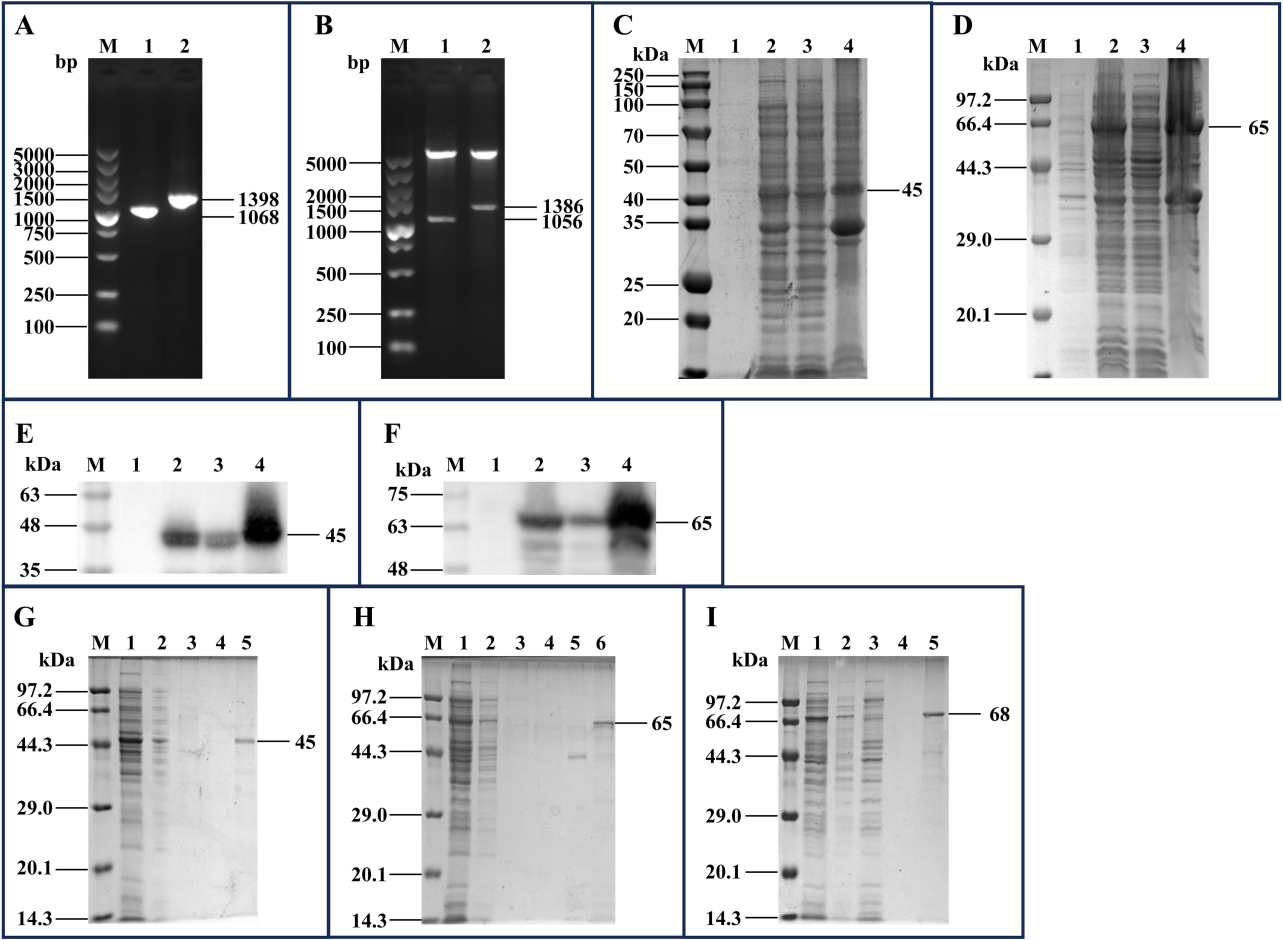
Cloning of the *mhp170* and *mhp274* genes, expression of the rMhp170 and rMhp274 proteins, and purification of the rMhp170, rMhp274, and rMhp336 proteins. **(A)** PCR amplification of the *mhp170* and *mhp274* genes by using the *E. coli* BL21(DE3)-pGEX-6P-1-mhp170 and *E. coli* BL21(DE3)-pGEX-6P-1- mhp274 strains as templates. Lane M: DNA marker; lane 1: PCR product composed of the *mhp170* gene, restriction enzyme sites of *Bam*HI and *Xho*I, and protective bases for *Bam*HI and *Xho*I; lane 2: PCR product composed of the *mhp274* gene, restriction enzyme sites for *Bam*HI and *Xho*I, and protective bases for *Bam*HI and *Xho*I. **(B)** Recombinant plasmids digested with *Bam*HI and *Xho*I. Lane M: DNA marker; lane 1: recombinant plasmid pET-30a(+)-mhp170 identified by digestion with *Bam*HI and *Xho*I; lane 2: recombinant plasmid pET-32a(+)-mhp274 identified by digestion with *Bam*HI and *Xho*I. **(C)** Identification of the expression of rMhp170 in recombinant bacteria by SDS−PAGE. **(D)** Identification of the expression of rMhp274 in recombinant bacteria via SDS−PAGE. **(E)** Identification of the expression of rMhp170 in recombinant bacteria via Western blotting with a mouse anti-His tag antibody. **(F)** Identification of the expression of rMhp274 in recombinant bacteria via Western blotting with a mouse anti-His tag antibody. **(C, E)** Lane M: protein marker; lane 1: total cell lysate of *E. coli* BL21 (DE3)-pET-30a(+)-mhp170 without IPTG induction; lane 2: total cell lysate of *E. coli* BL21 (DE3)-pET-30a(+)-mhp170 induced by IPTG; lane 3: the supernatant of the cell lysate of *E. coli* BL21 (DE3)-pET-30a(+)-mhp170 induced by IPTG; lane 4: the precipitate of the cell lysate of *E. coli* BL21 (DE3)-pET-30a(+)-mhp170 induced by IPTG. **(D, F)** Lane M: protein marker; lane 1: total cell lysate of *E. coli* BL21 (DE3)-pET-32a(+)-mhp274 without IPTG induction; lane 2: total cell lysate of *E. coli* BL21 (DE3)-pET-32a(+)-mhp274 induced by IPTG; lane 3: the supernatant of the cell lysate of *E. coli* BL21 (DE3)-pET-32a(+)-mhp274 induced by IPTG; lane 4: the precipitate of the cell lysate of *E. coli* BL21 (DE3)-pET-32a(+)-mhp274 induced by IPTG. **(G)** Recombinant Mhp170 protein purified by Ni^2+^ affinity chromatography. The supernatant of the cell lysate was loaded onto (lane 1) and allowed to flow through (lane 2) the column. The column was subsequently washed with a linear gradient of 0.1 mol/L (lane 3) and 0.2 mol/L (lane 4) imidazole, and the purified protein was collected (lane 5). **(H)** Recombinant Mhp274 protein purified by Ni^2+^ affinity chromatography. The supernatant of the cell lysate was loaded onto (lane 1) and allowed to flow through (lane 2) the column. The column was subsequently washed with a linear gradient of 0.01 mol/L (lane 3), 0.05 mol/L (lane 4) and 0.1 mol/L (lane 5) imidazole, and the purified protein was collected (Lane 6). **(I)** Recombinant Mhp336 protein purified by Ni^2+^ affinity chromatography. The supernatant of the cell lysate was loaded onto (lane 1) and allowed to flow through (lane 2) the column. The column was washed with 0.1 mol/L imidazole (lanes 3 and 4), and the purified protein was collected (lane 5).

The *mhp170* and *mhp274* genes were amplified via PCR (**Figure 2A**) and ligated to pET-30a(+) and pET-32a(+), respectively. The recombinant plasmids pET-30a(+)-mhp170 and pET-32a(+)-mhp274 were identified by digestion with *Bam*HI and *Xho*I (**Figure 2B**) and sequencing. The recombinant proteins Mhp170 (rMhp170) and Mhp274 (rMhp274) were expressed as both soluble proteins and in inclusion bodies, as confirmed by SDS−PAGE (**Figures 2C, D**) and Western blotting (**Figures 2E, F**). The recombinant proteins containing a 6 × His-tag at the C-terminus were successfully purified via a Ni^+^ affinity chromatography column (**Figures 2G, H**). Moreover, the recombinant protein Mhp336 (rMhp336) was also purified from *E. coli* BL21(DE3)-pET-32a(+)-mhp336 (**Figure 2I**).

### 3.2 Physical examination, body weights, AWGR and lung/body coefficient after immunization

After the first inoculation, all the mice exhibited smooth fur, a normal appetite, a desire to drink, and no neurological symptoms or allergies. The subunit vaccines were well absorbed, and there was no lump at the injection sites after the first and second inoculations. However, after the third immunization, some of the mice in the three experimental groups (three (25.0%) in the Mhp170 group, four (33.3%) in the Mhp274 group, and two (16.7%) in the Mhp336 group) exhibited unabsorbed lumps at the subcutaneous injection sites before necropsy. No mice died during the experiment.

**Figure 3:**
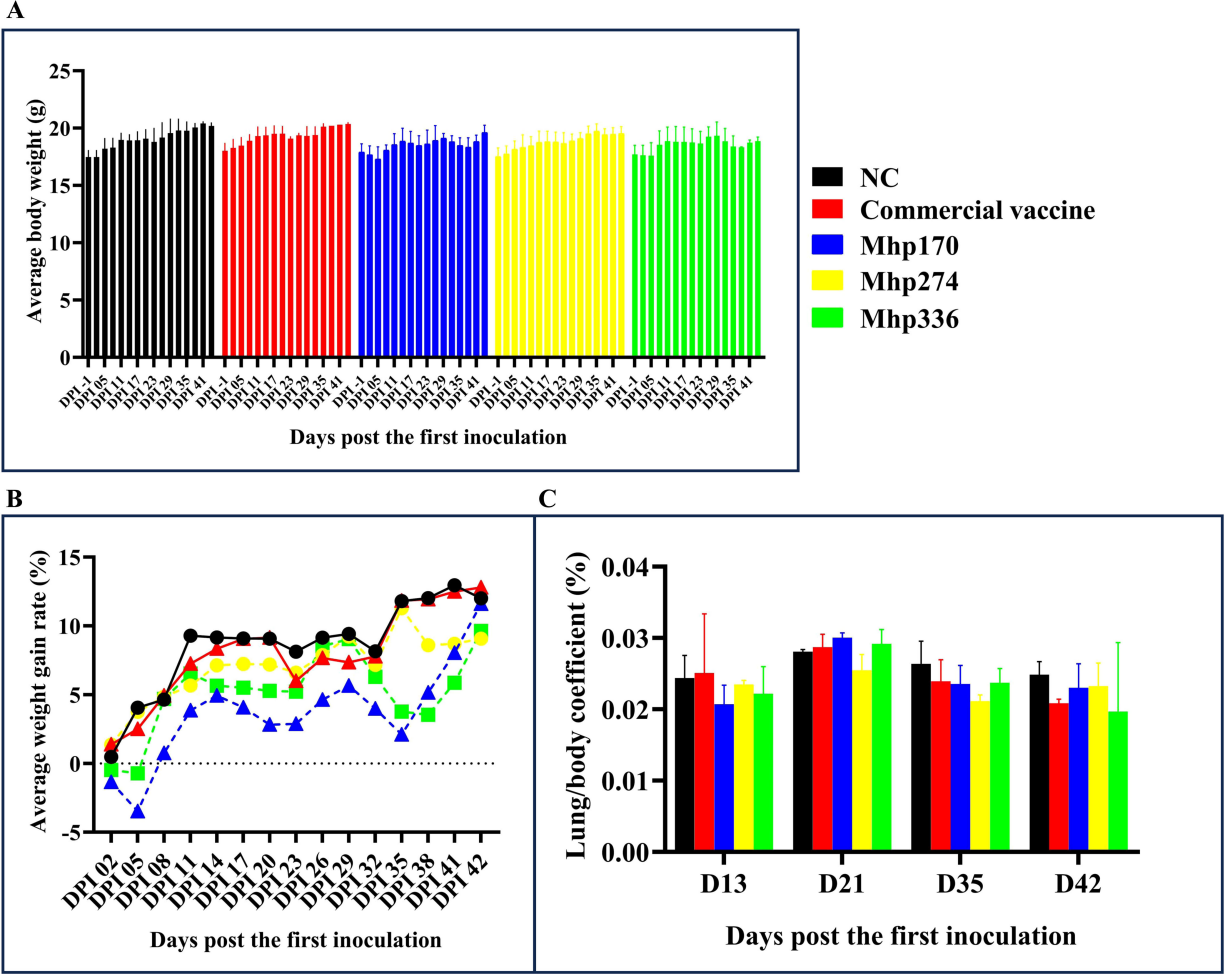
Body weights and lung/body coefficients after immunization. **(A)** Average body weight of the mice in the different groups on different days post the first inoculation. **(B)** The AWGRs of different groups of mice were calculated every three days post the first inoculation. **(C)** Changes in the lung/body coefficient in the different groups at 13, 21, 35, and 42 DPI.

Except for the decrease in average body weight observed in the rMhp170 and rMhp336 groups at 29–38 DPI, the average body weights of all the mice tended to increase throughout the experiment (**Figure 3A**). Overall, there was no significant difference in the average body weight among the mouse groups. The AWGR of the mice in each group is shown in **Figure 3B**. From 0 to 42 DPI, the AWGR of the mice in the NC and commercial vaccine groups was 12%, that in the Mhp170 group was 11%, and that in the Mhp274 and Mhp336 groups was 9%. However, by the end of the experiment, there was no significant difference in the AWGR among the groups.

The lung/body coefficients of the commercial vaccine, rMhp170 and rMhp336 groups were slightly greater than those of the NC group at 21 DPI, whereas at 35 DPI and 42 DPI, the lung/body coefficients of the commercial vaccine group and three experimental groups were lower than those of the NC group (**Figure 3C**). There was no significant difference among different groups.

### 3.3 Macroscopic and microscopic lung lesions

**Figure 4:**
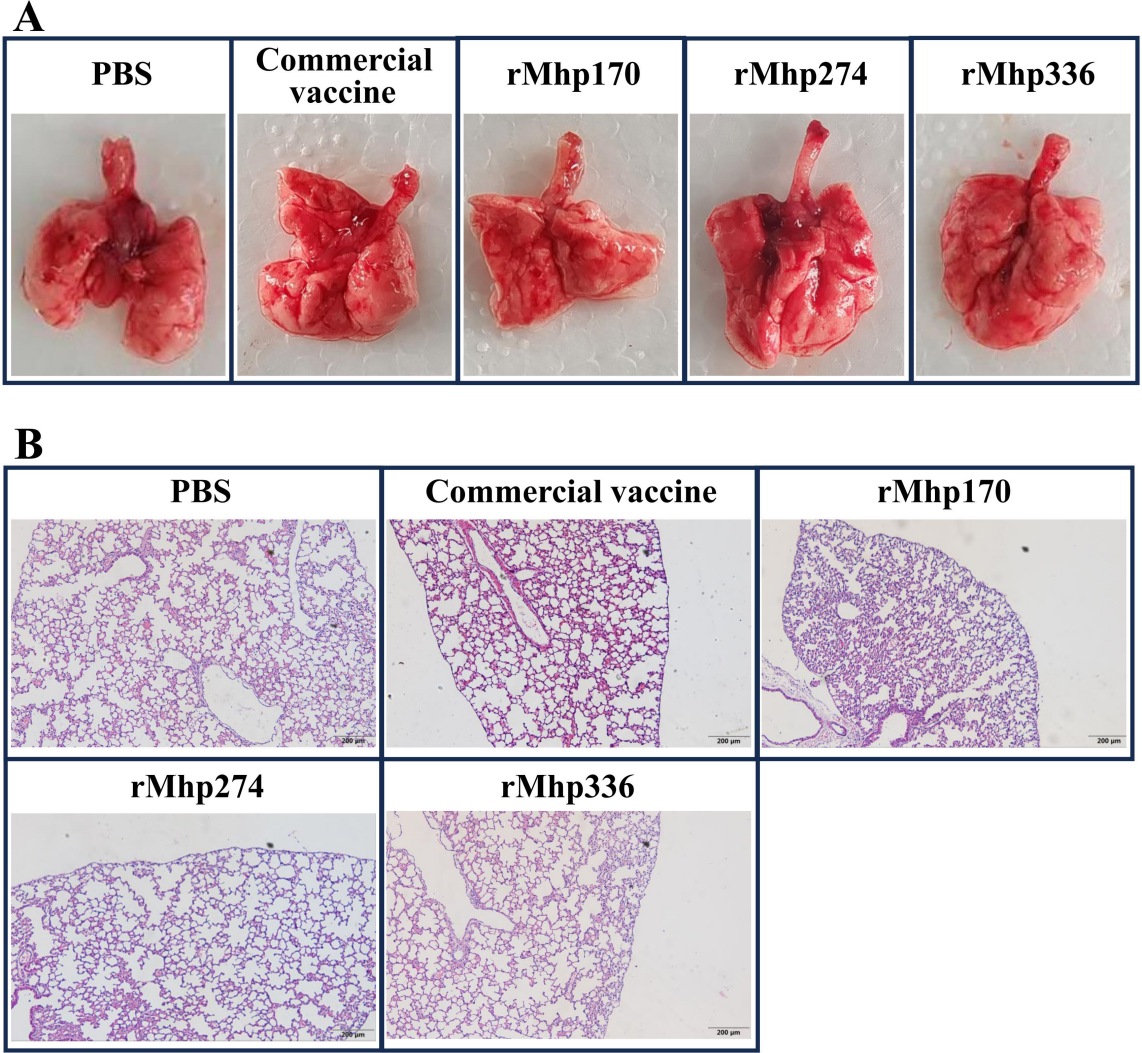
**(A)** Macroscopic assessment of lung lesions in different groups after immunization. **(B)** Microscopic analysis of pathological changes in cardiac lobes of the lungs of different groups after immunization (magnification,100 ×).

Macroscopic lung consolidation lesions and microscopic lung lesions were observed after the mice were euthanized. No significant gross lesions were found in the lungs of the mice in the NC group or all the immunized groups. However, there was slight bleeding in the lungs, which was caused by the collection of BALF from the mice before they were euthanized (**Figure 4A**). Afterward, histopathological lesions in the lungs were evaluated via H&E staining (**Figure 4B**). Similarly, there was slight bleeding in the bronchioles and alveolar cavities of mice in all the groups.

### 3.4 IgG, IgG1, and IgG2a in sera and BALF induced by the commercial vaccine and different recombinant proteins

**Figure 5:**
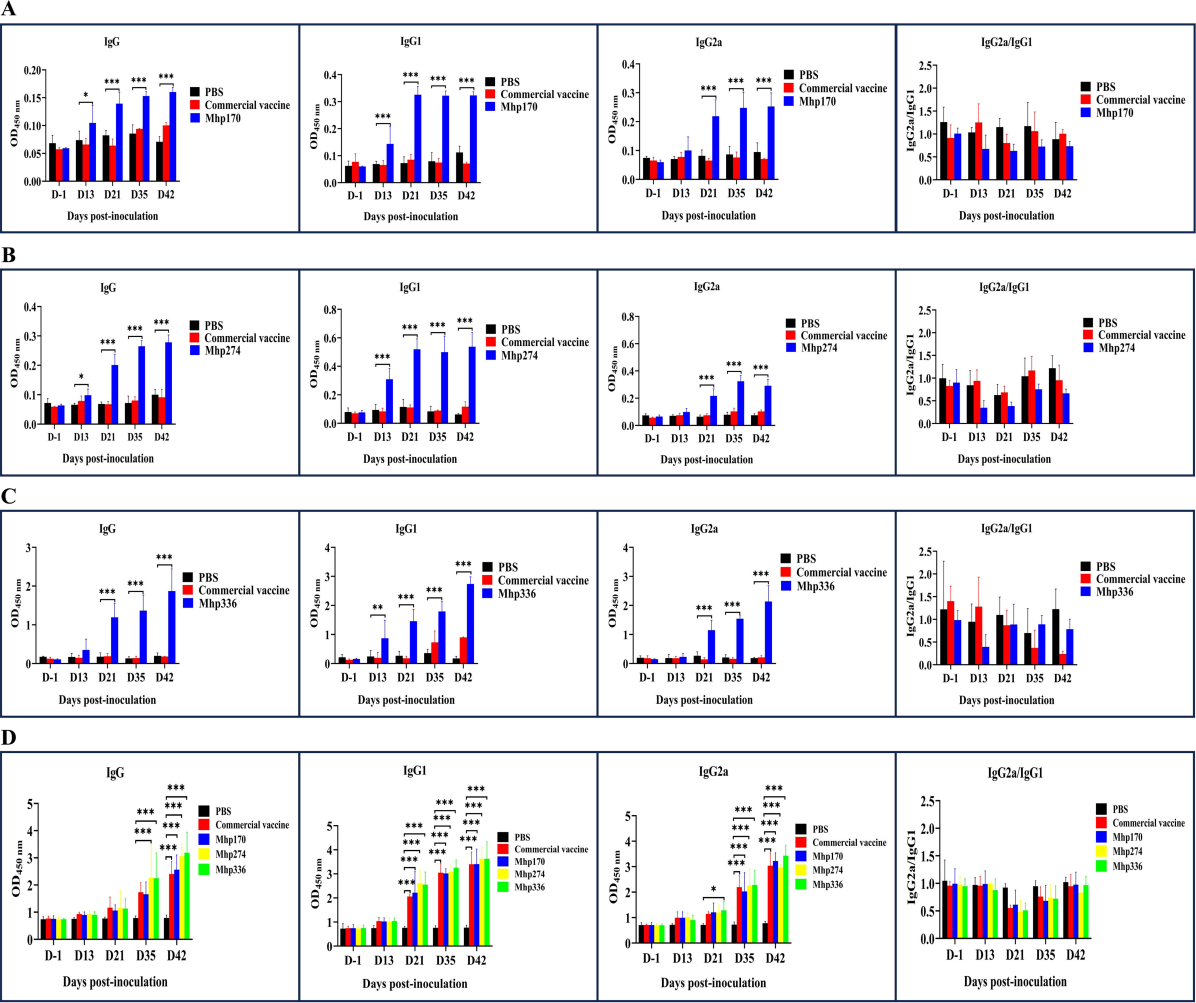
The levels of IgG and its isotypes in sera induced by the commercial vaccine and recombinant proteins. **(A)** The rMhp170-specific IgG antibodies and their isotypes were measured via rMhp170-coated ELISA plates. **(B)** The rMhp274-specific IgG antibodies and their isotypes were measured via rMhp274-coated ELISA plates. **(C)** The rMhp336-specific IgG antibodies and their isotypes were measured via rMhp336-coated ELISA plates. **(D)** IgG antibodies and their isotypes against commercial vaccine, rMhp170, rMhp274, or rMhp336 were measured via ELISA plates coated with crude extract from the J strain. The means ± SDs of 3 independent experiments are presented and were compared via one-way ANOVA; *p ≤ 0.05, **p ≤ 0.01, and ***p ≤ 0.001.

The strength of the host response to commercial vaccine, rMhp170, rMhp274, and rMhp336 was assessed by measuring the antigen-specific antibodies that responded to rMhp170, rMhp274, rMhp336, or the crude extract of the J strain in 96-well microtiter plates containing serum or BALF. IgG antibody levels increased significantly in sera against rMhp170 and rMhp274 after the first dose (13 DPI) and in sera against rMhp336 after the second dose (21 DPI) in corresponding recombinant protein-coated plates (**Figures 5A–C**). However, anti-commercial vaccine antibody levels did not increase significantly, as shown in the three recombinant protein-coated plates (**Figures 5A–C**). Moreover, the IgG antibodies levels against the crude extract of the J strain were detected in rMhp274 and rMhp336 groups from 35 DPI and for commercial vaccine and rMhp170 on 42 DPI (**Figure 5D**). These results indicated that immunization significantly increased the serum IgG titer in the immunized groups compared with that in the NC group.

The serum immune responses to commercial vaccine and the three recombinant proteins were further examined by measuring the levels of the IgG isotype subclasses IgG1 and IgG2a. In mice, IgG1 is an indicator of a Th2-type response, whereas IgG2a is predominant during a Th1-type response (Haddad et al., 1998). IgG1 antibodies against the three recombinant proteins were detected at 13 DPI and IgG2a antibodies were detected at 21 DPI with the corresponding recombinant protein-coated plates (**Figures 5A-C**). However, when whole cells of the J strain were used as the coating antigen, the levels of IgG1 against commercial vaccine and the three recombinant proteins and the levels of IgG2 against rMhp336 increased from 21 DPI, and the levels of IgG2 against commercial vaccine, rMhp170 and rMhp274 increased significantly from 35 DPI (**Figure 5D**). As shown in **Figure 5**, the levels of IgG2a were lower than those of IgG1 in the sera of the mice immunized with the three recombinant proteins, indicating that the three recombinant proteins could induce a mixed Th1/Th2 immune response, with Th2 being predominant.

IgG and its subtypes associated with three recombinant proteins and the crude extract of the J strain were also measured in BALF samples collected at different times after inoculation. The levels of IgG1 antibodies against rMhp170 increased significantly (*p* ≤ 0.001) from 35 DPI, and the level of IgG2a antibodies against rMhp170 increased significantly (*p* ≤ 0.01) on 42 DPI (**Supplementary Figure S1A**). Significant increases in the levels of IgG1 (*p* ≤ 0.001) and IgG2a (*p* ≤ 0.001), which were detected on rMhp274-coated plates, were detected on 21 DPI and from 35 DPI, respectively (**Supplementary Figure S1B**). The levels of IgG antibodies against rMhp336 were significantly greater on 13 DPI (*p* ≤ 0.001) (**Supplementary Figure S1C**). A significant increase in the levels of IgG and its subtypes against the crude extract of the J strain in the four immunized groups was not detected. The above results revealed that the levels and duration of the effects of IgG and its subtypes against recombinant proteins in BALF samples were much lower than those in sera.

### 3.5 IgA in BALFs induced by a commercial vaccine and different recombinant proteins

To determine whether the mice subcutaneously immunized with recombinant proteins developed mucosal immune responses, the level of specific sIgA antibodies in the BALF was measured via indirect ELISA (**Supplementary Figure S2**). The level of rMhp274-specific sIgA significantly increased on 42 DPI in Mhp274 and commercial vaccine groups (*p* ≤ 0.001) (**Supplementary Figure S2B**). Nevertheless, after immunization, there were no significant changes in the level of sIgA against rMhp170 or rMhp336 (**Supplementary Figure S2A, C**) compared with that in the NC group throughout the inoculation period. Similarly, no significant changes were observed in the levels of sIgA against commercial vaccine or the three recombinant proteins before and after immunization when the crude extract of the J strain was used as the coating antigen for the ELISA (**Supplementary Figure S2D**). The above results showed that three *M. hyopneumoniae* proteins induced only low levels of sIgA antibodies (Mhp274) or did not stimulate the mucosal immune response (Mhp170 and Mhp336) in BALF samples.

### 3.6 Cytokine expression in sera and splenocytes

**Figure 6:**
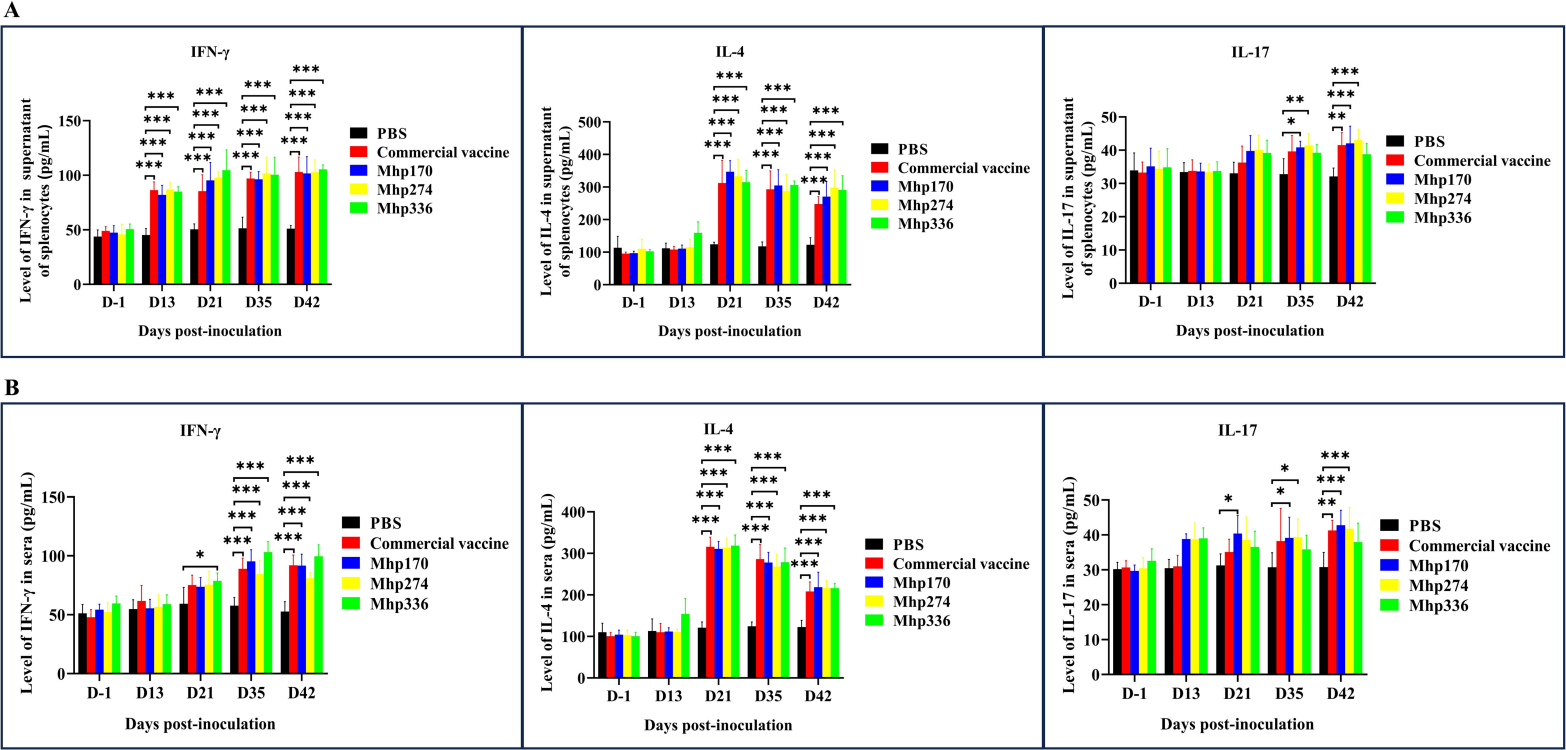
Cytokine levels in the supernatants of splenocytes and sera. **(A)** Cytokine levels in the supernatant of splenocytes. **(B)** Cytokine levels in sera. The means ± SDs of 3 independent experiments are presented and were compared via one-way ANOVA; *p ≤ 0.05, **p ≤ 0.01, and ***p ≤ 0.001.

To further evaluate the types of immune responses (Th1, Th2, and Th17), indirect ELISA was used to compare IFN-γ, IL-4, and IL-17 production in the supernatants of splenocytes stimulated with recombinant proteins and in sera of immunized animals. The commercial vaccine and three vaccine candidate proteins stimulated splenocytes (**Figure 6A**) to produce high levels of IFN-γ from 13 DPI. The production of IFN-γ in sera was detected in the rMhp336 group after 21 DPI and in the commercial vaccine, rMhp170 and rMhp274 groups after 35 DPI (**Figure 6B**). Moreover, the commercial vaccine and three vaccine candidate proteins stimulated both the splenocytes (**Figure 6A**) and sera (**Figure 6B**) of the mice to produce high levels of IL-4 from 21 DPI. However, rMhp170 stimulated the mice to produce high levels of IL-17 in splenocytes from 35 DPI (**Figure 6A**) and in sera from 21 DPI (**Figure 6B**), and IL-17 was detected in the supernatant of splenocytes and in the sera of the mice stimulated with rMhp274 from 35 DPI (**Figure 6**). Moreover, the level of IFN-γ was lower than the level of IL-4 in both the splenocyte and serum supernatants, indicating that rMhp170, rMhp274, and rMhp336 could induce a Th2-biased immune response. This finding is consistent with the detection results for IgG1. In addition, the cytokine mRNA levels in the splenocyte population were analyzed via qPCR (**Supplementary Figure S3**). The expression of IFN-γ mRNA induced by rMhp170, rMhp274 and rMhp336 was significantly increased at 42 DPI. Moreover, the expression of IL-4 mRNA was promoted at 42 DPI after stimulation with the commercial vaccine, and at 35 DPI after stimulation with rMhp170, rMhp274, and rMhp336. However, the expression of IL-17 mRNA was elevated on 42 DPI by immunization with rMhp170 and rMhp274. These results indicated that the commercial vaccine and three candidate proteins induced both humoral (Th1-associated) and cellular (Th2-associated) immune responses, but rMhp336 could not effectively stimulate the IL-17-type cellular immune response. In addition, these results demonstrated that the protein expression of cytokines was not associated with mRNA expression and that antigen stimulation promoted an increase in cytokine protein expression in the body earlier than increased cytokine mRNA expression.

### 3.7 Lymphocyte proliferation after stimulation

**Figure 7:**
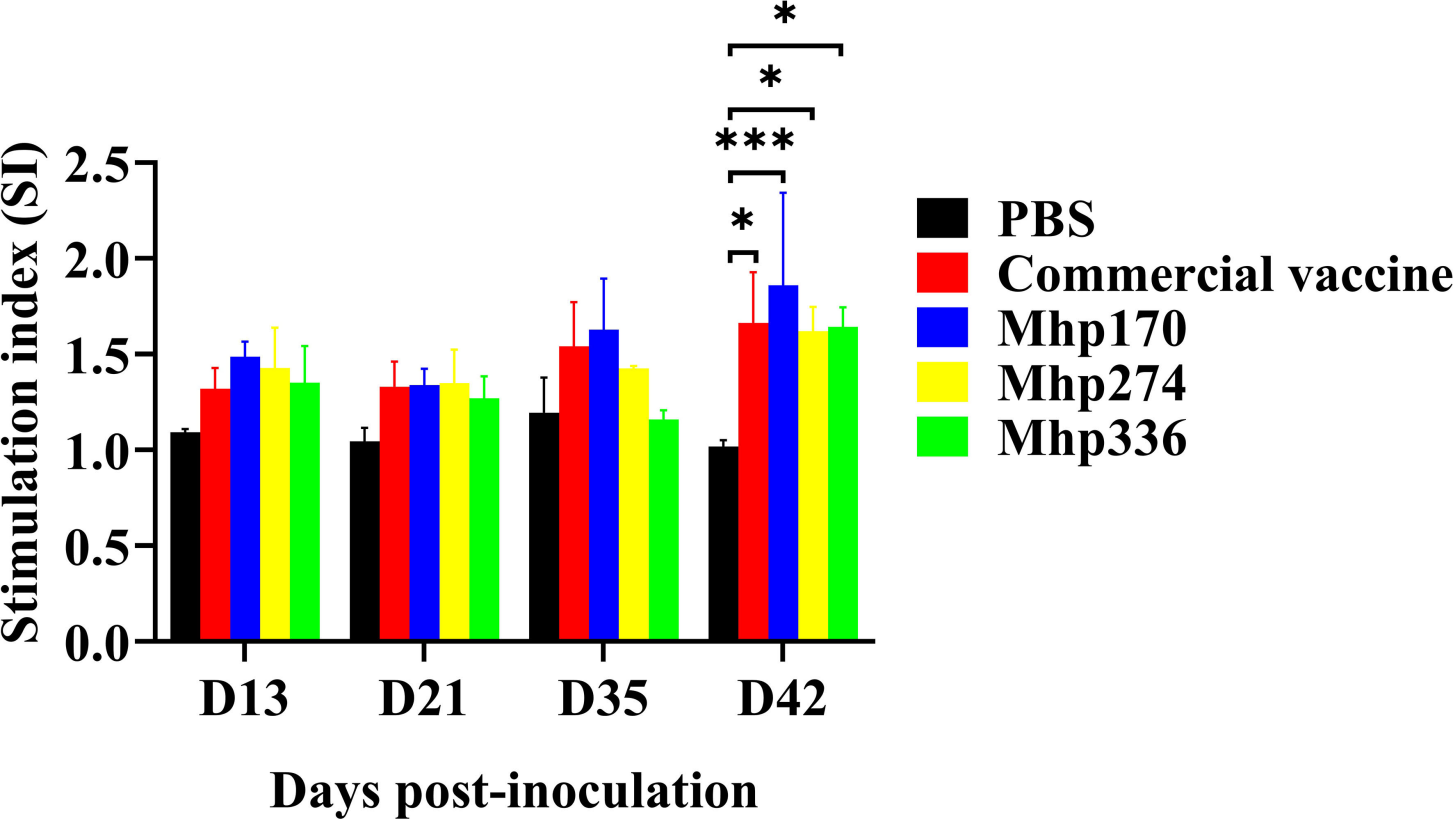
Lymphocyte proliferation assays in mice immunized with commercial vaccine or recombinant proteins. Spleen lymphocytes were isolated at 13 DPI, 21 DPI, 35 DPI, and 42 DPI. Recombinant proteins were administered to stimulate lymphocytes. The SI values were compared between the NC group and the immunized groups. The means ± SDs of 3 independent experiments are presented and were compared via one-way ANOVA; *p ≤ 0.05, and ***p ≤ 0.001.

To further determine the effects of the recombinant proteins on the cellular immune response, spleen lymphocytes were isolated at 13 DPI, 21 DPI, 35 DPI, and 42 DPI and stimulated with the commercial vaccine or the recombinant proteins. The lymphocyte proliferation responses were measured, and the SI values are shown in **Figure 7**. There was no difference from 13 DPI to 35 DPI among the NC, commercial vaccine and experimental groups. However, at 42 DPI, the levels of SI in the commercial vaccine group and three experimental groups were significantly greater than those in the NC group. These findings implied that the three *M. hyopneumoniae* vaccine candidate proteins could stimulate the cellular immune response.

## 4 Discussion

Owing to the widespread prevalence of *M. hyopneumoniae* on swine farms (Maes et al., 2018) and its ability to hide within host cells (Deeney et al., 2019; Wen et al., 2021), the implementation of management strategies and the administration of antimicrobials have been ineffective in controlling the spread of *M. hyopneumoniae*. Vaccination is the most commonly used measure for controlling MPS. The major advantages of *M. hyopneumoniae* vaccination are related to improvements in daily weight gain (2–8%) and the feed conversion ratio (2–5%). Additionally, a shorter time to reach slaughter weight, fewer clinical signs, fewer lung lesions and lower treatment costs have been reported (Maes et al., 1999). Despite their beneficial effects, these bacterins provide only partial protection and do not prevent the colonization of epithelial cells by *M. hyopneumoniae* (Arsenakis et al., 2019; Meyns et al., 2006; Sibila et al., 2008; Villarreal et al., 2011b) or the transmission of *M. hyopneumoniae* from vaccinated pigs to nonvaccinated pigs (Meyns et al., 2006; Villarreal et al., 2011a). Commercial vaccines (including attenuated live and inactivated adjuvanted vaccines) have not achieved the expected immunity because of their inability to stimulate both systemic and mucosal immune responses in the host (Martelli et al., 2014; Shen et al., 2017). The bottleneck in the development of vaccines for MPS is the stimulation of effective and comprehensive immune responses in the host. Several *M. hyopneumoniae* proteins, such as P97 (Marchioro et al., 2014a; Okamba et al., 2010), P42 (Jorge et al., 2014; Marchioro et al., 2014a), and NrdF (Marchioro et al., 2014a), have been evaluated for their ability to elicit host immune responses. Mhp274 is one of paralogs of P102 (Minion et al., 2004), and its characteristics have not been reported. Mhp170 and Mhp336 are two hypothetical proteins annotated in *M. hyopneumoniae* genomes (Minion et al., 2004; Vasconcelos et al., 2005; Liu et al., 2011). However, previous studies have shown that these proteins were humoral immunodominant proteins that can strongly react with *M. hyopneumoniae*-positive porcine sera (Ning et al., 2020, 2023). Mhp170 and Mhp274 had reactions with all 11 tested *M. hyopneumoniae*-positive sera (Ning et al., 2023), and Mhp336 was recognized by 10 of the 11 positive sera (Ning et al., 2020). In this study, we evaluated the capacity of these proteins to elicit both systemic and mucosal immune responses in mice.

After immunization, significant levels of specific IgG antibodies were produced in the sera of the three experimental groups, indicating that vaccine candidate proteins could evoke humoral immune responses in mice. Some studies have demonstrated that *M. hyopneumoniae*-specific IgG levels in serum and BALF are not correlated with protection (Djordjevic et al., 1997; Thacker et al., 1998). However, an increasing number of studies have shown that specific IgG play an important role in resisting the pathogenicity conferred by *M. hyopneumoniae* proteins. For example, antisera against Mhp597 could inhibit the nuclease activity of rMhp597 (Li et al., 2022), and sera from rAdP97c (a recombinant adenovirus vector designed to express the C-terminal portion of the *M. hyopneumoniae* P97 adhesin)-vaccinated pigs inhibited the growth of *M. hyopneumoniae* in a dose-dependent manner (Okamba et al., 2010). The growth of *M. hyopneumoniae* was inhibited by the antiserum of P42, and the inhibitory effect was concentration dependent (Chen et al., 2003). Moreover, in slaughter-aged pigs, both the percentage of lungs with cranioventral pulmonary consolidation and the mean lung lesion score were significantly greater in *M. hyopneumoniae* seropositive farms than on seronegative farms (Garcia-Morante et al., 2017). Therefore, the humoral immune response induced by *M. hyopneumoniae* and its proteins in the host cannot be ignored.

The adherence of *M. hyopneumoniae* to the mucosal surface of the respiratory tract is the crucial step in the establishment of infection (Blanchard et al., 1992; DeBey and Ross, 1994). SIgA is the major contributor to mucosal immunity and plays a crucial role in the defense against pathogens (Park et al., 2024). Mucosal immunity appears to play an important role in the control of *M. hyopneumoniae* infection (Ferreira et al., 2023). The ability to stimulate the production of sIgA in the body is increasingly emphasized in the development of *M. hyopneumoniae* vaccines. In several studies, the production of sIgA in pigs has been stimulated by adding mucosal immune adjuvants to *M. hyopneumoniae* vaccines. SBA-15-containing oral vaccines decrease the shedding of *M. hyopneumoniae* and lead to mucosal protection, as confirmed by the reduction in pulmonary lesions and prevention of invasion and adherence of *M. hyopneumoniae* (Ferreira et al., 2023; Mechler-Dreibi et al., 2021). Several studies have shown that *M. hyopneumoniae* proteins fused to the *E. coli* heat-labile enterotoxin B subunit, a potent mucosal and parenteral adjuvant, have the ability to induce specific IgA in BALF, with statistically significant differences from the control group (Marchioro et al., 2014a, 2014b). Additionally, the intradermal administration of an adjuvanted bacterin was demonstrated to enhance the local humoral immune response induced by commercial vaccines (Martelli et al., 2014). The delivery of protein antigens through delivery vehicle vectors can also enhance mucosal immune responses. For example, the level of saliva P97c-specific IgA in rAdP97c-vaccinated pigs was significantly greater than that in inactivated vaccine-inoculated pigs, and the average daily weight gain was slightly greater in the rAdP97c-vaccinated pigs than in the unvaccinated and challenged pigs. Moreover, the production of IgA induced by the rAdP97c vaccine in pigs occurred earlier than the production of IgA induced by the commercial inactivated vaccine in saliva (Okamba et al., 2010). P97 has also been delivered orally or intranasally with several bacterial vectors, such as *Bacillus subtilis* (Wang et al., 2019), *Erysipelothrix rhusiopathiae* (Ogawa et al., 2009);, and *Salmonella* Choleraesuis (Zhou et al., 2022), and strong levels of P97-specific sIgA were observed in BALF and/or sera. After challenge with the *M. hyopneumoniae* strain, the results revealed that these bacterial vector-encapsulated proteins alleviated weight loss and reduced clinical symptoms and the severity of pneumonic lung lesions (Ogawa et al., 2009; Zhou et al., 2022). The immunization with recombinant bacterial vectors expressing P42 (*Salmonella* Choleraesuis) (Zhou et al., 2022) or P46 (*Bacillus subtilis*) (Wang et al., 2019) also induced strong mucosal immune responses and stimulated significant production of sIgA in BALF and/or sera, which improved the average daily weight gain and reduced lung lesions (Zhou et al., 2022). In the present study, rMhp274 emulsified with Freund’s complete/incomplete adjuvant induced the secretion of specific sIgA in BALF at 42 DPI compared with that in the NC group. Unfortunately, there was no significant increase in sIgA in the rMhp170 and rMhp336 groups compared with the NC group. Freund’s complete adjuvant mainly induces Th1-type immune responses, whereas Freund’s incomplete adjuvant mainly induces Th2-type immune responses (Shibaki and Katz, 2002). However, these two adjuvants have a weaker ability to induce mucosal immunity (Jang et al., 2011). Moreover, both adjuvants are not absorbed well. After the third immunization, some of the mice in the experimental groups exhibited unabsorbed lumps at the subcutaneous injection sites. Therefore, to enhance mucosal immune responses and stimulate animals to secrete sIgA earlier and reduce undesirable side effects, efforts will be made in the future to immunize animals with candidate vaccine proteins coexpressed with antigen delivery vehicle vectors or mixed with mucosal and systemic immune adjuvants.

It has been proposed that the cellular immune response plays an important role in enhancing protective immunity against *M. hyopneumoniae* infection (Thacker et al., 2000; Okamba et al., 2010), 51]. CD4^+^ T cells play a major role in coordinating immune responses (Zhu et al., 2010). On the basis of their distinct populations of cytokine profiles, CD4^+^ T cells can be classified into Th1, Th2 or Th17 cells (Luckheeram et al., 2012; Korn et al., 2009). Th1 cells are involved in cell-mediated immune responses and activate B cells to produce opsonizing antibodies, such as IgG2a, whereas Th2 cells contribute to humoral immunity, promoting the secretion of IgG1 and IgA (Zhou et al., 2022). Th17 cells produce IL-17, IL-17A, and IL-22, thereby inducing a massive tissue reaction owing to the broad distribution of the IL-17 and IL-22 receptors (Korn et al., 2009). IFN-γ, IL-4 and IL-17 are the effector cytokines produced by Th1, Th2 and Th17 cells, respectively (Luckheeram et al., 2012; Korn et al., 2009). Our results demonstrated that rMhp170 and rMhp274 were capable of inducing the secretion of IFN-γ, IL-4 and IL-17 in sera and BALF, the mRNA expression of IFN-γ, IL-4 and IL-17 in splenocytes, and the production of high levels of SI. Nevertheless, rMhp336 only induces secretion of IFN-γ and IL-4 in serum and BALF, mRNA expression of IFN-γ and IL-4 in splenocytes, and stimulation of lymphocyte proliferation. The above results illustrated that Mhp170 and Mhp274 induced systemic Th1/Th2/Th17 immune responses and that Mhp336 induced a mixed Th1/Th2-type immune response.

In this study, we explored the mucosal and systemic immune responses of three humoral immunodominant proteins of *M. hyopneumoniae* in BALB/c mice, but some issues still need to be clarified in subsequent research. First, how the level of the local humoral immune response can be further increased remains to be determined. Second, how high levels of sIgA can be induced in the respiratory mucosa at an earlier stage remains to be determined. We will subsequently attempt to immunize animals with candidate vaccine proteins coexpressed with delivery vehicle vectors or mixed with mucosal and systemic immune adjuvants to enhance local humoral and mucosal immune responses. It is much to be regretted that we have not yet established a mouse model of *M. hyopneumoniae* infection. Fortunately, we have isolated dozens of strains of *M. hyopneumoniae*. We will use these strains to establish a mouse model of *M. hyopneumoniae* infection and further evaluate the immunoprotective effects of these proteins in mice.

## 5 Conclusion

In conclusion, we successfully expressed and purified three *M. hyopneumoniae* immunodominant proteins. After immunization with the recombinant purified proteins, there were no significant adverse reactions or macroscopic or microscopic lung lesions in the mice. Mhp170 and Mhp274 induced systemic Th1/Th2/Th17 immune responses, and Mhp274 increased the level of sIgA in BALF at 42 DPI. However, Mhp336 only stimulated the mice to produce mixed Th1/Th2-type immune responses. The protective effects of the recombinant proteins on mice and pigs need to be further evaluated in subsequent research.

## Data Availability

The original contributions presented in the study are included in the article/**Supplementary Material**. Further inquiries can be directed to the corresponding author/s.

## Supplementary material

Supplementary Figure 1 The levels of IgG and its isotypes in BALF induced by commercial vaccine and recombinant proteins. **(A)** The rMhp170-specific IgG antibodies and their isotypes were measured via rMhp170-coated ELISA plates. **(B)** The rMhp274-specific IgG antibodies and their isotypes were measured via rMhp274-coated ELISA plates. **(C)** The rMhp336-specific IgG antibodies and their isotypes were measured via rMhp336-coated ELISA plates. **(D)** IgG antibodies and their isotypes against commercial vaccine, rMhp170, rMhp274, or rMhp336 were measured via ELISA plates coated with crude extracts from the J strain. The means ± SDs of 3 independent experiments are presented and were compared via one-way ANOVA; **p* ≤ 0.05, ***p* ≤ 0.01, and ****p* ≤ 0.001.

Supplementary Figure 2 The levels of IgA in BALF induced by commercial vaccine and recombinant proteins. **(A)** The rMhp170-specific IgA antibodies were measured via rMhp170-coated ELISA plates. **(B)** The rMhp274-specific IgA antibodies were measured via rMhp274-coated ELISA plates. **(C)** The rMhp336-specific IgA antibodies were measured via rMhp336-coated ELISA plates. **(D)** IgA antibodies against commercial vaccine, rMhp170, rMhp274, or rMhp336 measured via ELISA plates coated with crude extracts of the J strain. The means ± SDs of 3 independent experiments are presented and were compared via one-way ANOVA; **p* ≤ 0.05, ***p* ≤ 0.01.

Supplementary Figure 3 Cytokine expression in splenocytes was analyzed by qPCR. The means ± SDs of 3 independent experiments are presented and were compared via one-way ANOVA; **p* ≤ 0.05, ***p* ≤ 0.01, and ****p* ≤ 0.001.
