# Supplementary figures and images for: The Mycoplasma hyopneumoniae protein Mhp274 elicits mucosal and systemic immune responses in mice
Source: Front Cell Infect Microbiol. 2025 Feb 7;15:1516944. doi: 10.3389/fcimb.2025.1516944 (PMC11842358; doi:10.3389/fcimb.2025.1516944)

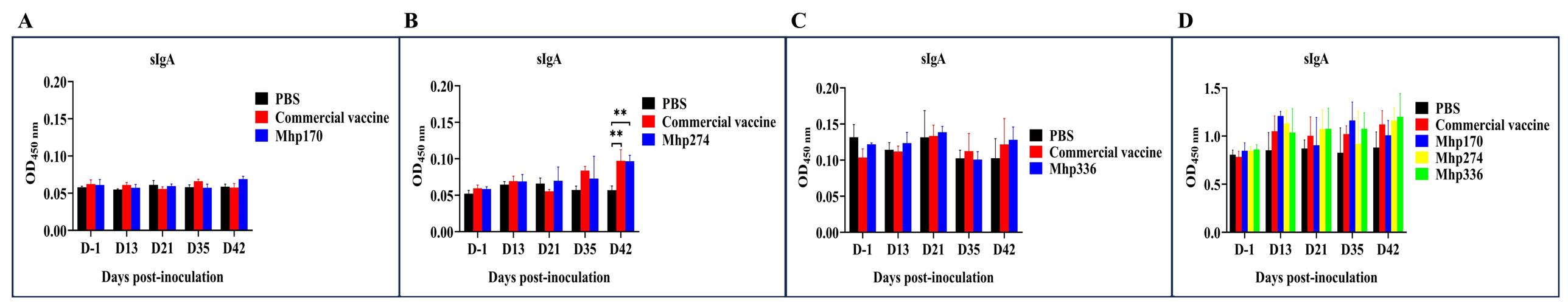

Supplement: Supplementary Figure 2 — The levels of IgA in BALF induced by commercial vaccine and recombinant proteins. (A) The rMhp170-specific IgA antibodies were measured via rMhp170-coated ELISA plates. (B) The rMhp274-specific IgA antibodies were measured via rMhp274-coated ELISA plates. (C) The rMhp336-specific IgA antibodies were measured via rMhp336-coated ELISA plates. (D) IgA antibodies against commercial vaccine, rMhp170, rMhp274, or rMhp336 measured via ELISA plates coated with crude extracts of the J strain. The means ± SDs of 3 independent experiments are presented and were compared via one-way ANOVA; *p ≤ 0.05, **p ≤ 0.01. [file Image2.jpeg]

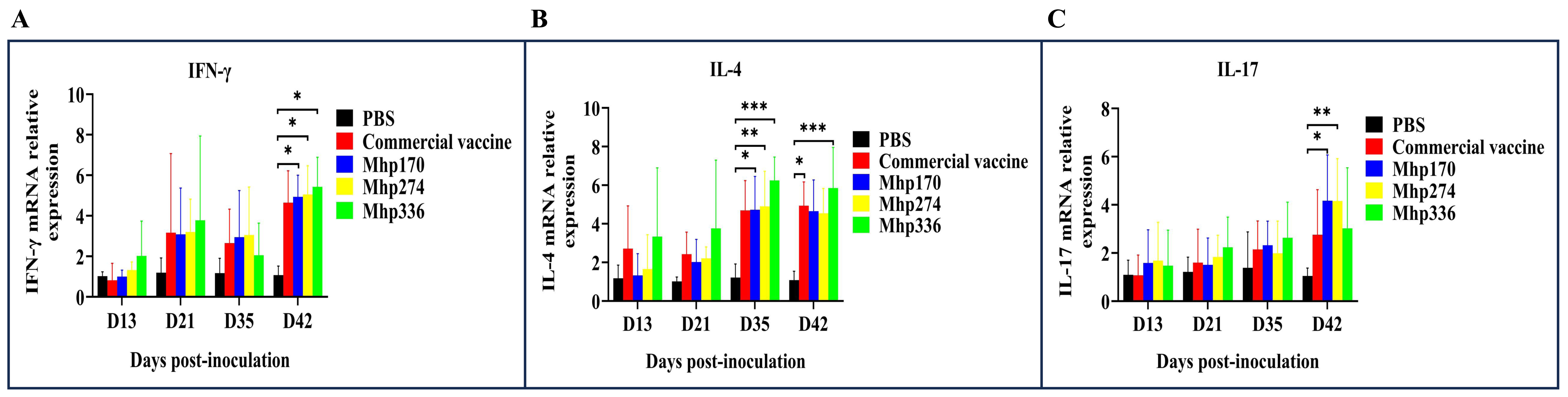

Supplement: Supplementary Figure 3 — Cytokine expression in splenocytes was analyzed by qPCR. The means ± SDs of 3 independent experiments are presented and were compared via one-way ANOVA; *p ≤ 0.05, **p ≤ 0.01, and ***p ≤ 0.001. [file Image3.jpeg]
