# Supplementary material for: The Mycoplasma hyopneumoniae protein Mhp274 elicits mucosal and systemic immune responses in mice
Source: Front Cell Infect Microbiol. 2025 Feb 7;15:1516944. doi: 10.3389/fcimb.2025.1516944 (PMC11842358; doi:10.3389/fcimb.2025.1516944)
Supplement: Supplementary file 4 [file Table1.docx]

Supplementary Material

**TABLE S1.** Primers used for amplification of the *mhp170* and *mhp274* genes of *M. hyopneumoniae*.

| Gene | Primer | Primer sequence (5'‒3') | Annealing temperature |
| --- | --- | --- | --- |
| *mhp170* | mhp174-F | CGCGGATCCATGTTGAAAAAAAAATTTAGAAAAATTT | 55℃ |
|  | mhp174-R | CCGCTCGAGATTTTTTTCGTTAAACACGTATT |  |
| *mhp274* | mhp274-F | CGCGGATCCATGAAGTTAGCAAAATTACTTAAA | 55℃ |
|  | mhp274-R | CCGCTCGAGTGCATCTTGATCTTCAGGCAT |  |

The protective bases are underlined with a wavy line; the restriction enzyme sites of *Bam*HI and *Xho*I are underlined with a straight line.
