# Supplementary material for: The Mycoplasma hyopneumoniae protein Mhp274 elicits mucosal and systemic immune responses in mice
Source: Front Cell Infect Microbiol. 2025 Feb 7;15:1516944. doi: 10.3389/fcimb.2025.1516944 (PMC11842358; doi:10.3389/fcimb.2025.1516944)
Supplement: Supplementary file 5 [file Table2.docx]

Supplementary Material

**TABLE S2.** Primers used for amplification of *IFN-γ*, *IL-4* and *IL-17*.

| Gene | Primer | Primer sequence (5'‒3') | Annealing temperature | Reference |
| --- | --- | --- | --- | --- |
| *IFN-γ* | IFN-γ-F | TCAAGTGGCATAGATGTGGAAGAA | 60℃ | Wang et al., 2019 |
|  | IFN-γ-R | TGGCTCTGCAGGATTTTCATG |  |  |
| *IL-4* | IL-4-F | ACAGGAGAAGGGACGCCAT | 60℃ | Wang et al., 2019 |
|  | IL-4-R | GAAGCCCTACAGACGAGCTCA |  |  |
| *IL-17* | IL-17-F | CTGATCAGGACGCGCAAAC | 60℃ | Liu et al., 2020 |
|  | IL-17-R | TCGCTGCTGCCTTCACTGTA |  |  |
| *β-actin* | β-actin-F | CCCTAAGGCCAACCGTGAA | 60℃ | Wang et al., 2019 |
|  | β-actin-R | CAGCCTGGATGGCTACGTACA |  |  |
